# Toward a comprehensive language for biological systems

**DOI:** 10.1186/1741-7007-9-68

**Published:** 2011-10-17

**Authors:** James R Faeder

**Affiliations:** 1 Department of Computational and Sytems Biology, University of Pittsburgh School of Medicine, Pittsburgh, PA 15260 USA

## Abstract

Rule-based modeling has become a powerful approach for modeling intracellular networks, which are characterized by rich molecular diversity. Truly comprehensive models of cell behavior, however, must address spatial complexity at both the intracellular level and at the level of interacting populations of cells, and will require richer modeling languages and tools. A recent paper in *BMC Systems Biology *represents a signifcant step toward the development of a unified modeling language and software platform for the development of multi-level, multiscale biological models.

See research article: http://www.biomedcentral.com/1752-0509/5/166

## Modeling for biologists?

In his essay 'Can a biologist fix a radio?' molecular biologist Yuri Lazebnik highlighted the absurdity of some kinds of informal reasoning that pervade biology, and called for the development of biologist-accessible (if not exactly friendly) languages to promote more formal approaches to reasoning about and prediction of the behavior of molecular networks inside cells [1]. Although he suggested that the rise of systems biology might force biologists to change quickly, it is still a safe bet nearly a decade later that most experimental biologists are unlikely to be familiar with modeling and related software tools, let alone using them. This is despite the rapid rise of genomics and bioinformatics that has made the use of bioinformatics tools, such as BLAST, an essential part of training and practice.

**Figure 1:**
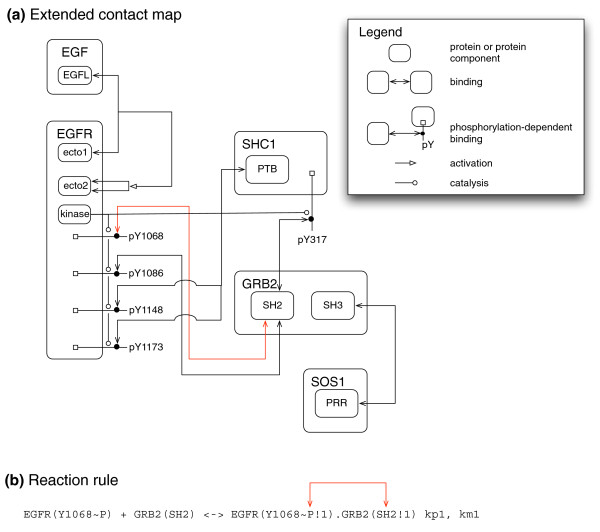
**Rule-based model of early signaling events mediated by the epidermal growth factor receptor (EGFR)**. **(a) **Global view of the model with an Extended Contact Map (ECM) (see [7] for more detailed explanation of the notation), which shows protein components - catalytic domains and sites of binding and postranslational modification - and their interactions. **(b) **Translation of one interaction (shown in red) from the ECM into a reaction rule in a rule-based model. The rule indicates that a specific tyrosine residue on EGFR (Y1048) must be phosphorylated in order for GRB2 to bind through its SH2 domain. The absence of other components in the rule indicates that the rate of binding is not affected by the status of other components of either protein - in other words, this rule neglects cooperative or allosteric effects.

I think that the development of rule-based modeling languages and tools, such as BioNetGen [2] and Kappa [3], in recent years represents a near-fulfillment of Lazebnik's vision of precise formal modeling languages for biology, at least at the molecular level. Modeling of biochemical networks is plagued by the problem of combinatorial complexity, which is the explosion in the number of possible species and reactions that may occur among molecules that have multiple components [4]. In conventional approaches to modeling reaction kinetics, such as ordinary differential equations, each species and reaction must be explicitly represented in the model - either entered into a file or drawn with a computer program. The basic building blocks of a rule-based model, on the other hand, are structured objects or graphs. Various types of graphs are used and the general term 'site graph' has been proposed to refer to them [5]. In a site graph, vertices represent material components of proteins, such as sites of binding and chemical modification. Rules, which are composed of site graphs, describe interactions among components in a precise way (Figure 1). Because the rule-based modeling approach is based on a graphical formalism, it is easy to visualize models and link formal model elements to the material components being represented (Figure 1a) [6]. The domains and motifs of a signaling protein correspond to vertices of a site graph, which provides a representation of the protein analagous to the graphs used to represent atomic structures in chemistry. Rules describe the interactions among model elements referring only to the sites that are involved and without reference to sites that are not (Figure 1b). This is similar to the way reactions are described in organic chemistry, where the parts of a molecule that do not participate in a reaction are left unspecified [5]. Combinatorial complexity can thus be avoided in the specification of a model as long as most interactions are local, that is, they involve only a few of the possible sites of the interacting molecules, which is a reasonable assumption based on current knowledge.

Because of the development of general-purpose rule-based languages and simulators, it is now possible to construct biochemical models of an arbitrary number of network components at a high level of resolution and to simulate the model in a reasonable amount of time on a desktop computer [3,7,8]. Many challenges remain, not the least of which are making the tools more accessible to bench biologists and, perhaps more important, fostering a culture in which modeling is more commonly used as a reasoning aid. In the near future I envision that biologists will be able to construct models using tools very similar to those that are used to search the literature and online knowledge bases, and they will be able to use these models to predict the outcome of possible experiments and to gain insight into the possible mechanism through which the predicted effect may arise. Even researchers with limited mathematical or computational experience should be able to engage fully in the productive cycle of experimentation followed by modeling followed by further experimentation.

To summarize up to this point, rule-based modeling now provides a scalable way to model the complex molecular biochemistry that is employed by cells to process information. Incorporating such models into everyday study of signaling systems could have a profound impact on molecular biology. So far, however, I have considered only what goes on inside cells when they are treated as well-mixed chemical bags, and not their internal organization or how they interact with each other, which of course is fundamental to biology. Furthermore, a fundamental challenge in biology, to understand the genetic basis of phenotype, requires coupling predictive models of intracellular biochemistry with models of higher levels of organization - cells, tissues, organs, and so on - in a bi-directional way. Since its inception, however, systems biology has been more oriented toward the molecular, intracellular level, which is reflected in the fact that most of the modeling tools that have been developed are aimed at the development of chemical network models and do not provide capabilities for constructing models that span multiple levels of resolution. For example, standardized exchange formats for systems biology models, such as Systems Biology Markup Language (SBML) [9], do not readily support such embedding.

## 'Leveling up'

**Figure 2:**
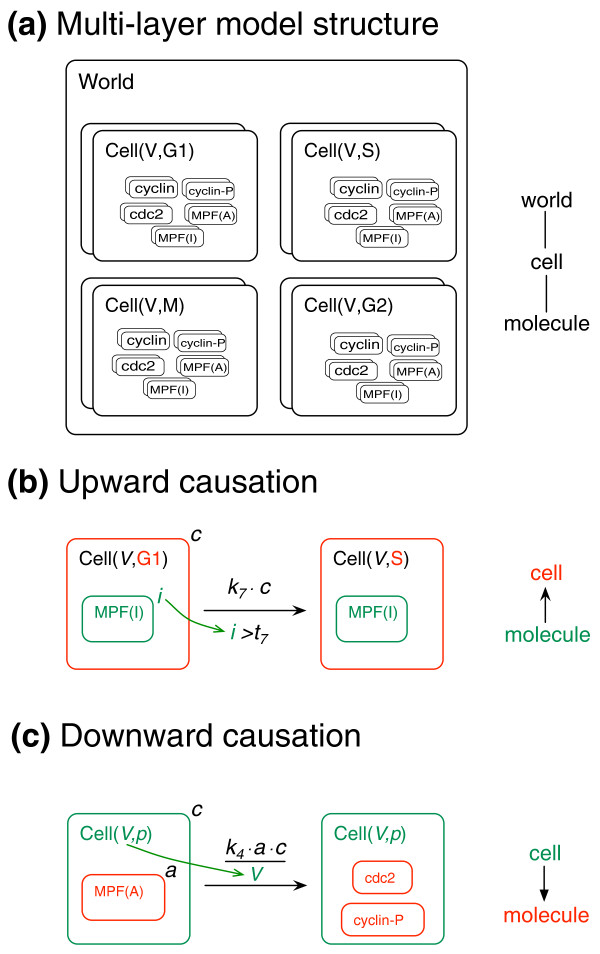
**Multi-level rule-based model of yeast cell cycle regulation (after **[10]**)**. **(a) **Nested view of the model structure. The world node contains a population of cells, each of which has two attributes, volume and cell cycle state. Stacking of boxes representing each entity indicates a variable number of instances. Each cell contains a population of molecules that comprise the biochemical components of the cell cycle. **(b) **Upward causation - components of the molecular layer influence dynamics of the cell layer. The number of MPF molecules in the I state (for 'inactive') controls the passage from G1 to S. The formula beneath the arrow must be true in order for the rule to fire. **(c) **Downward causation - the state of the cell influences the rate of a biochemical transformation of contained molecules.

Maus *et al*. [10] have addressed a growing need for modeling tools that span multiple biological levels of organization by developing a multi-level rule-based language, called ML-Rules. As in other rule-based languages, structured objects represent proteins and their components, but they may also represent higher levels of organization, such as organelles and cells. The key extension in comparison to other rule-based languages is that objects may contain collections of other objects, and this embedding relationship can affect the behavior of both container and contents (Figure 2). For example, a cell may contain a collection of molecules representing the regulatory components of the cell cycle (Figure 2a), and progression of the cell through the cell cycle can be coupled to the collective properties of the cell cycle network, that is, the level of active maturation promoting factor (MPF) (Figure 2b). This is an example of upward causation, in which properties of the lower level components affect the behavior of the higher level. In the other direction, properties of the cell, such as its volume, can affect the reaction rates of the enclosed network (Figure 2c). This is an example of downward causation.

Both forms of causation may be concisely represented in ML-Rules (Figure 2b, c), allowing for the specification of multi-level models. For example, a population of interacting cells may be modeled as a collection of cells, each of which contains a collection of molecules that interact via globally defined rules. The movement, growth, and division of cells may be defined by rules that act at the cell level, whereas binding, uptake, and secretion of molecules may be defined by rules that span the cell and molecular levels. The description of the intracellular level could be further refined by inclusion of such processes as endocytosis and nuclear import/export, which would also require additional levels of representation for endosomes, nucleus, and so on.

ML-Rules is the first fully implemented rule-based modeling language that has been described in the literature and is capable of integrating detailed molecular biochemistry into multi-level models. The hierarchical representation used in ML-Rules is related to a more general formulation called reactive bigraphs, which also uses a nested object hierarchy and reaction rules to represent the interactions that can take place in a complex network [11]. Several biological languages based on reactive bigraphs have been proposed (for example, [12]), but software implementations have so far not been presented.

There are, however, other general-purpose tools available for the integration of rule-based biochemistry, as described above, into multi-level models (for example, [13,14]). These tools use different mathematical and computational models to describe the dynamics at each level of the system, and can in this sense be termed 'heterogeneous'. Of these, the most accessible for a general audience is probably the Simmune platform, which has a graphical interface that integrates all stages of modeling from model construction to data analysis and allows embedding of rule-based biochemical descriptions into cellular agents [14]. There are also other general purpose tools for multi-level modeling; an example is CompuCell3D [15], which allows reaction networks described in SBML to be embedded in cellular simulations of varying sophistication but cannot yet handle rule-based specifications of the biochemistry.

The cell cycle example presented by Maus *et al*. could probably be implemented in each of the heterogeneous tools mentioned, as well as others. Each of these implementations, however, would likely be more difficult to understand and less flexible than the corresponding ML-Rules implementation because of the lack of a unifying language and adherence to a pre-defined level hierarchy. In most of the current frameworks, models are specified in the form of plain-text files and/or high-level program code in languages such as Python and C++. The embedding of levels is either fixed or achieved through calls to specific functions in a programming library. ML-Rules, on the other hand, provides a formal biological language for expressing all parts of the model. The number of levels and the physical model for simulating each level can be achieved by refactoring the rules.

The flexibility of ML-Rules does come with a cost, however. Describing higher-level processes such as cell division with rules requires some sophistication on the part of the modeler; it is not simply a matter of translating knowledge about a specific molecular interaction into a rule. Such barriers could be overcome by defining rule templates that a modeler can use for specific types of behavior and creating libraries, but it remains to be seen whether the heterogeneous approaches mentioned above or the unified approach taken by ML-Rules provide a better basis for the development of intuitive modeling tools for the biologist. Simulation efficiency is also an issue that needs to be addressed before more realistic applications are possible. The stochastic simulation algorithm implemented in the current version of ML-Rules is limited to relatively small populations of cells. Although no direct performance comparisons have been carried out, heterogeneous simulators, which usually have highly optimized simulators, are probably capable of performing much larger-scale simulations on the same system.

## In search of the Killer App

What is needed for dynamical systems modeling of the type enabled by tools discussed here to take off among experimental biologists? Lowering the barrier to using tools and to using existing knowledge to create models is clearly a key requirement. At the level of molecular biochemistry, rule-based modeling represents a key conceptual advance, although much work needs to be done to make it more broadly accessible. Languages for describing multi-level models are going to take more work and time because of the inherent complexity of the challenge, in terms of both representation and simulation. Finding the right balance of flexiblity and simplicity is difficult.

What is probably more critical for wider adoption, however, is the demonstration that these types of models can lead to new discoveries that could not otherwise be made - a 'Killer App'. It could take the form of a model that a large community of biologists adopts for the study of a specific system - for example, yeast pheremone signaling, cell cyle, or bacterial chemotaxis. Such an example could be instrumental in convincing biologists to make rule-based modeling part of their standard toolkit for fixing radios.
